# Supplementary material for: Cell cycle progression mechanisms: slower cyclin-D/CDK4 activation and faster cyclin-E/CDK2
Source: bioRxiv. 2023 Aug 17:2023.08.16.553605. Preprint. [Version 1] doi: 10.1101/2023.08.16.553605 (PMC10542123; doi:10.1101/2023.08.16.553605)
Supplement: Supplement 1 [file media-1.pdf]

Supplementary Material:
Cell cycle progression mechanisms: slower cyclin-D/CDK4 activation and faster cyclin-E/CDK2

Wengang Zhang, Yonglan Liu, Hyunbum Jang, and Ruth Nussinov*

* Ruth Nussinov
Email: NussinoR@mail.nih.gov

This PDF file includes:

Figures S1 to S5
Tables S1

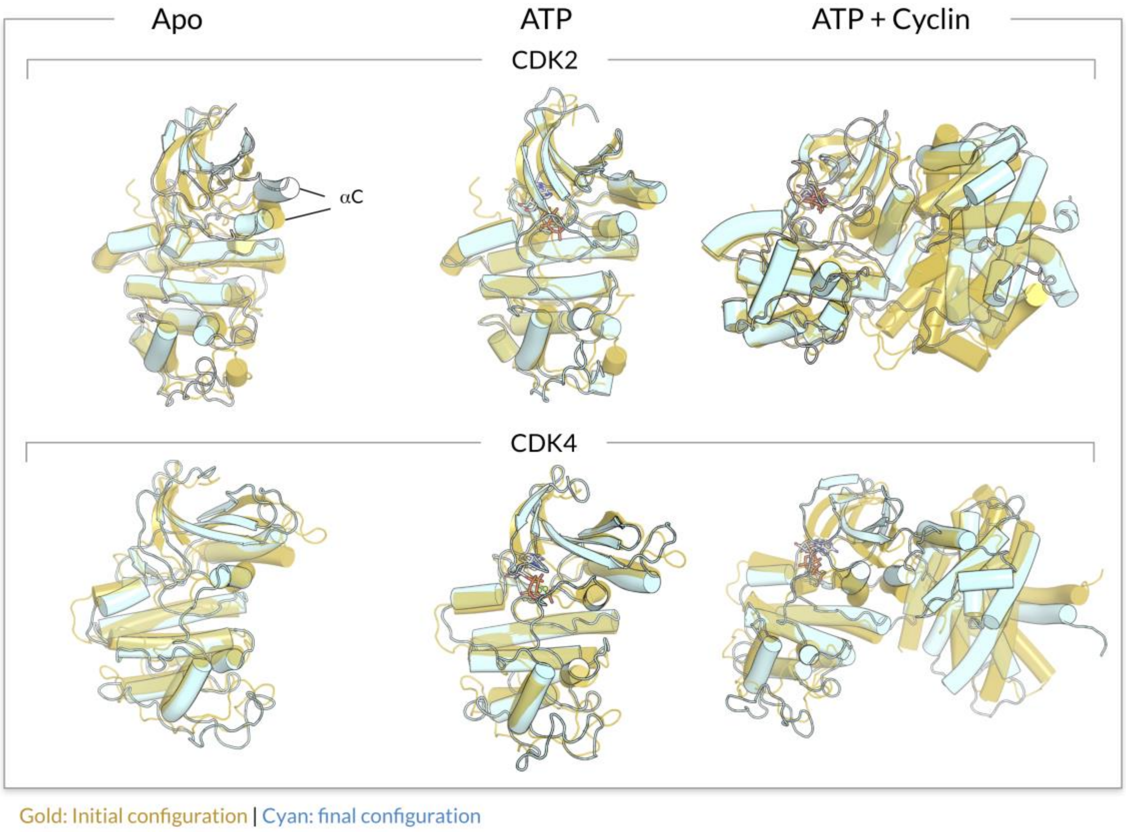

Fig. S1. Superimposition of the final conformation (cyan cartoon) into the starting point (gold cartoon) for the CDK2 (*upper panel*) and CDK4 (*lower panel*) systems. From left to right column, the cartoons are represented in the following order: inactive CDK2 and CDK4 monomer ("Apo"), inactive CDK2 and CDK4 monomer with ATP ("ATP"), and inactive CDK2 and CDK4 with ATP and their respective cyclin partners ("ATP + Cyclin").

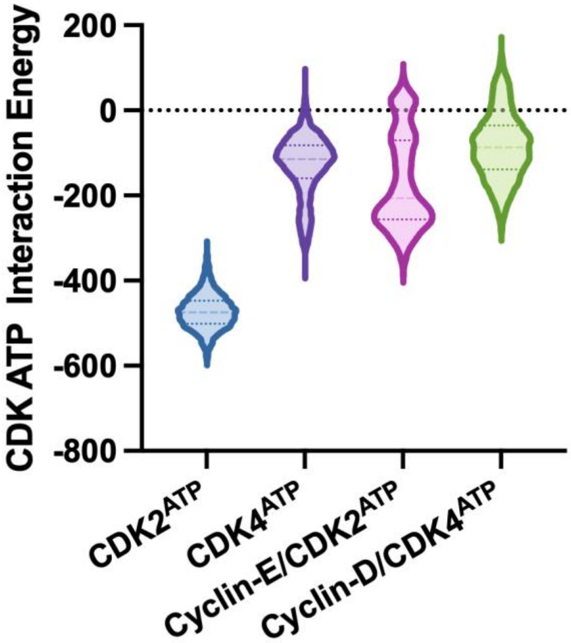

Fig. S2. Violin plots representing the interaction energy of ATP with the kinase domain of CDKs for the monomeric systems, $CDK2^{ATP}$ and $CDK4^{ATP}$, and the complexes, cyclin-E/$CDK2^{ATP}$ and cyclin-D/$CDK4^{ATP}$. In the monomeric CDKs, ATP exhibits a stronger interaction with $CDK2^{ATP}$ than with $CDK4^{ATP}$. For the complexes, ATP has a slightly stronger binding affinity to the CDK2 complex compared to the CDK4 complex.

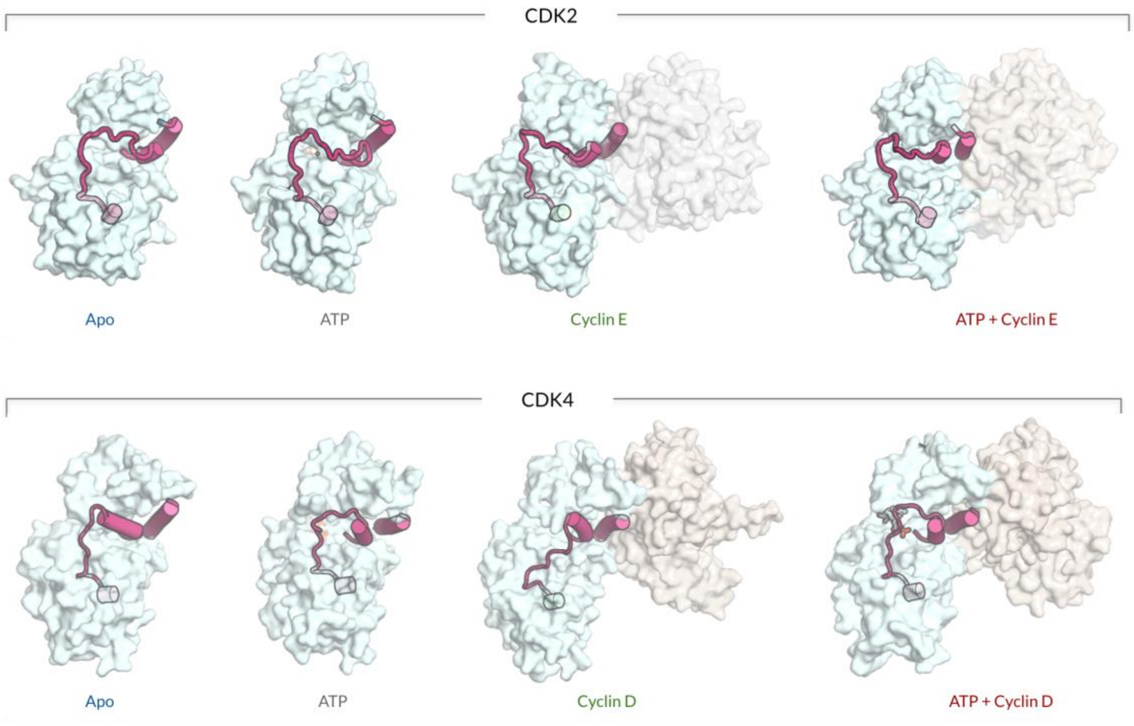

Fig. S3. Simulation snapshots highlighting the position of the αC-helix and A-loop. For CDK2, ATP loading or cyclin-E binding induces the outward movement of the A-loop (perpendicular to the page) and the inward movement of the αC-helix. In contrast, for CDK4, ATP loading or cyclin-D binding induces the movement of the inhibitory αL12-helix on the activation loop closer to the αC helix, while the position of the αC-helix remains relatively unchanged.

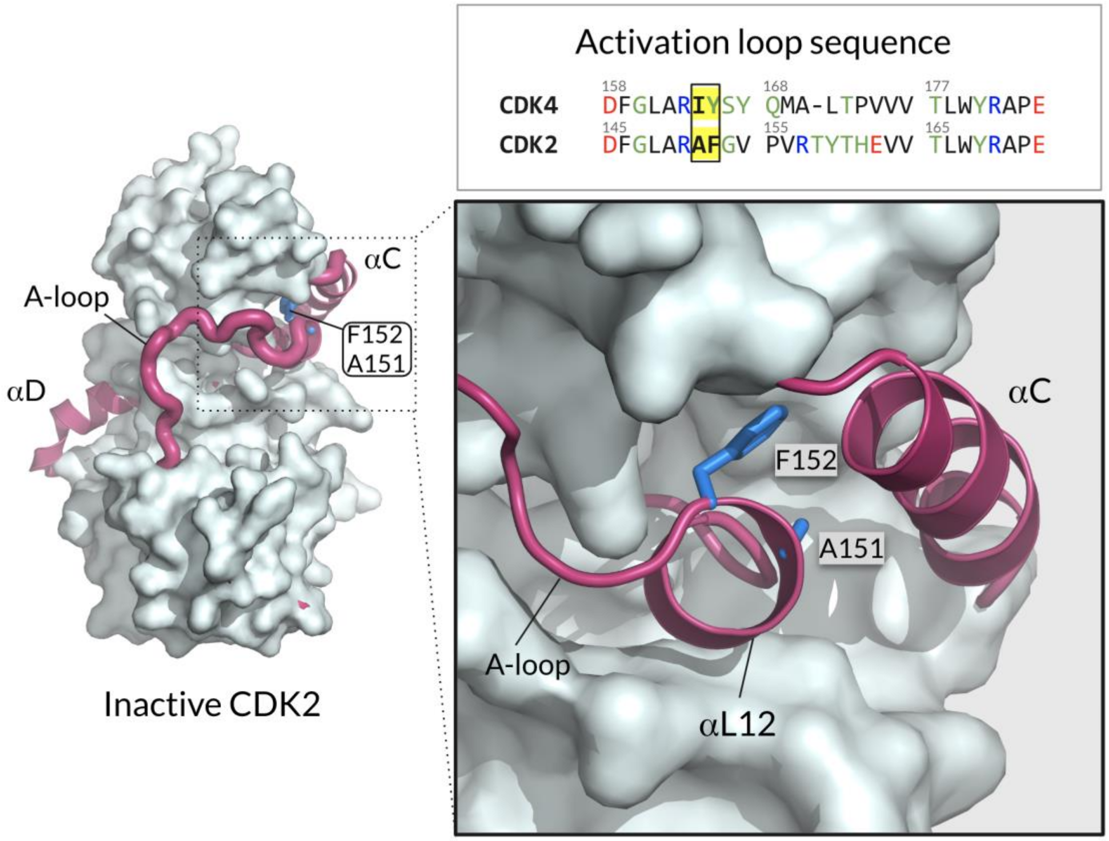

Fig. S4. Surface representation of the monomeric inactive CDK2^{Apo} with its A-loop and αC-helix rendered as thick tube and helix, respectively (*left panel*). Highlight (*right panel*) showing the position of the αL12-helix and αC-helix. F152 and A151 on the A-loop of CDK2 are also shown.

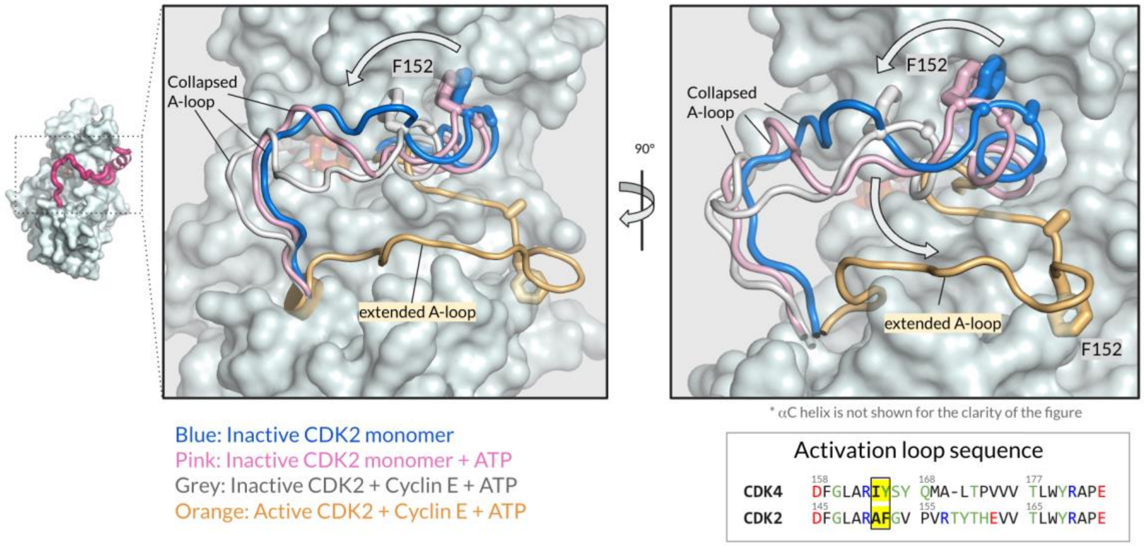

Fig. S5. Surface representation of the inactive CDK2^{Apo} with its A-loop and αC-helix rendered as thick tube and helix, respectively (*left panel*). Superimposition of the A-loop of the CDK2 systems (*middle and right panels*). It shows the movement of the A-loop of CDK2 upon ATP loading or cyclin-E binding, or both. We observe that the A-loop of CDK2 moves outward on either ATP loading or cyclin binding.

Table S1. Summary of simulation systems of the CDK2 and CDK4.

PDB	Systems	Num. of Atoms	Simulation Time (μs)
1HCL	$CDK2^{Apo}$	73387	2
1HCK	$CDK2^{ATP}$	73360	2
1W98 + 1HCL	Cyclin-E/$CDK2^{Apo}$	134582	2
1W98 + 1HCK	Cyclin-E/$CDK2^{ATP}$	134635	2
2W9Z	$CDK4^{Apo}$	73360	2
2W9Z	$CDK4^{ATP}$	73413	2
2W9Z	Cyclin-D/$CDK4^{Apo}$	134407	2
2W9Z	Cyclin-D/$CDK4^{ATP}$	134355	2
